# Chalcogenide phase-change material advances programmable terahertz metamaterials: a non-volatile perspective for reconfigurable intelligent surfaces

**DOI:** 10.1515/nanoph-2023-0645

**Published:** 2024-01-29

**Authors:** Kai Chen, Wenju Song, Zhaolin Li, Zihao Wang, Junqing Ma, Xinjie Wang, Tao Sun, Qinglei Guo, Yanpeng Shi, Wei-Dong Qin, Aimin Song, Hou-Tong Chen, Yifei Zhang

**Affiliations:** Shandong Technology Center of Nanodevices and Integration, School of Integrated Circuits, Shandong University, Jinan, 250100, China; Department of Critical Care Medicine, Qilu Hospital, Cheeloo College of Medicine, Shandong University, Jinan, 250100, China; Department of Electrical and Electronic Engineering, University of Manchester, Manchester, M13 9PL, UK; Center for Integrated Nanotechnologies, Los Alamos National Laboratory, Los Alamos, NM, 87545, USA; Institute of Novel Semiconductors, Shandong University, Jinan, 250100, China

**Keywords:** 6G, terahertz, Ge_2_Sb_2_Te_5_, non-volatile, reconfigurable intelligent surface

## Abstract

Terahertz (THz) waves have gained considerable attention in the rising 6G communication due to their large bandwidth. However, the cost and power consumption become the major constraints for the commercialization of 6G THz systems as the frequency increases. Reconfigurable intelligent surface (RIS) comprising active metasurfaces and digital controllers has been proposed for beamforming in the 6G multiple-input-multiple-output systems, showing good potential to suppress the system size, weight, and power consumption (SWaP). Currently, their controlling diodes can hardly work up to THz frequencies. Therefore, several active stimuli have been investigated as alternatives. Among them, chalcogenide phase-change material Ge_2_Sb_2_Te_5_ (GST) addresses large modulation depth, picosecond switching speed, and non-volatile properties. Notably, the non-volatile GST may enable RIS systems with memory and low control power. This work briefly reviews the advances of GST-tuned THz metamaterials (MTMs), discusses the current obstacles to overcome, and gives a perspective of GST applications in the rising 6G communications.

## 1 Introduction

As the global deployment of 5G technology advances, the telecommunications sector is steadily transitioning to 6G [1], [2], which is expected to dramatically enhance the key performances, such as data rates, energy efficiency, latency, and network density. This cutting-edge technology stands out for its promise of ultra-high data transmission rate up to several hundred Gbps and requires 10–100 times better energy efficiency than 5G [3]. It aims to harness a broader spectrum, focusing on terahertz (THz) frequencies, to realize unprecedented data transmission speeds and interconnected intelligence [4], [5]. THz waves, ranging from 0.1 to 10 THz, can offer expansive bandwidth and inherent robustness against interference, which is pivotal for achieving terabit-level data speeds in 6G networks [6]. However, integrating these technologies poses substantial challenges, particularly the high cost and power consumption associated with adapting multiple input multiple output (MIMO) systems from 5G to 6G, which could significantly escalate the price for each gigabit data. This highlights the urgent need for novel solutions that are both cost-effective and energy-efficient.

Reconfigurable intelligent surface (RIS) represents a promising technology with tremendous potential to address these challenges. By using digitally programmable metamaterials (MTMs), RIS dynamically controls electromagnetic waves, facilitating a versatile and manageable wireless transmission environment [7]. This innovative approach effectively reduces the hardware and financial demands of the traditional transmitters and receivers. Currently, RIS has been frequently investigated with positive-intrinsic-negative (PIN) and varactor diodes, however, which typically work well below 15 GHz. In addition, multilayer circuits are desired to bias these diodes individually [8]. Recently, optically controlled diodes, eliminating the need for multilayer circuits, have also demonstrated promising potential in RIS applications [9].

**Figure 1:**
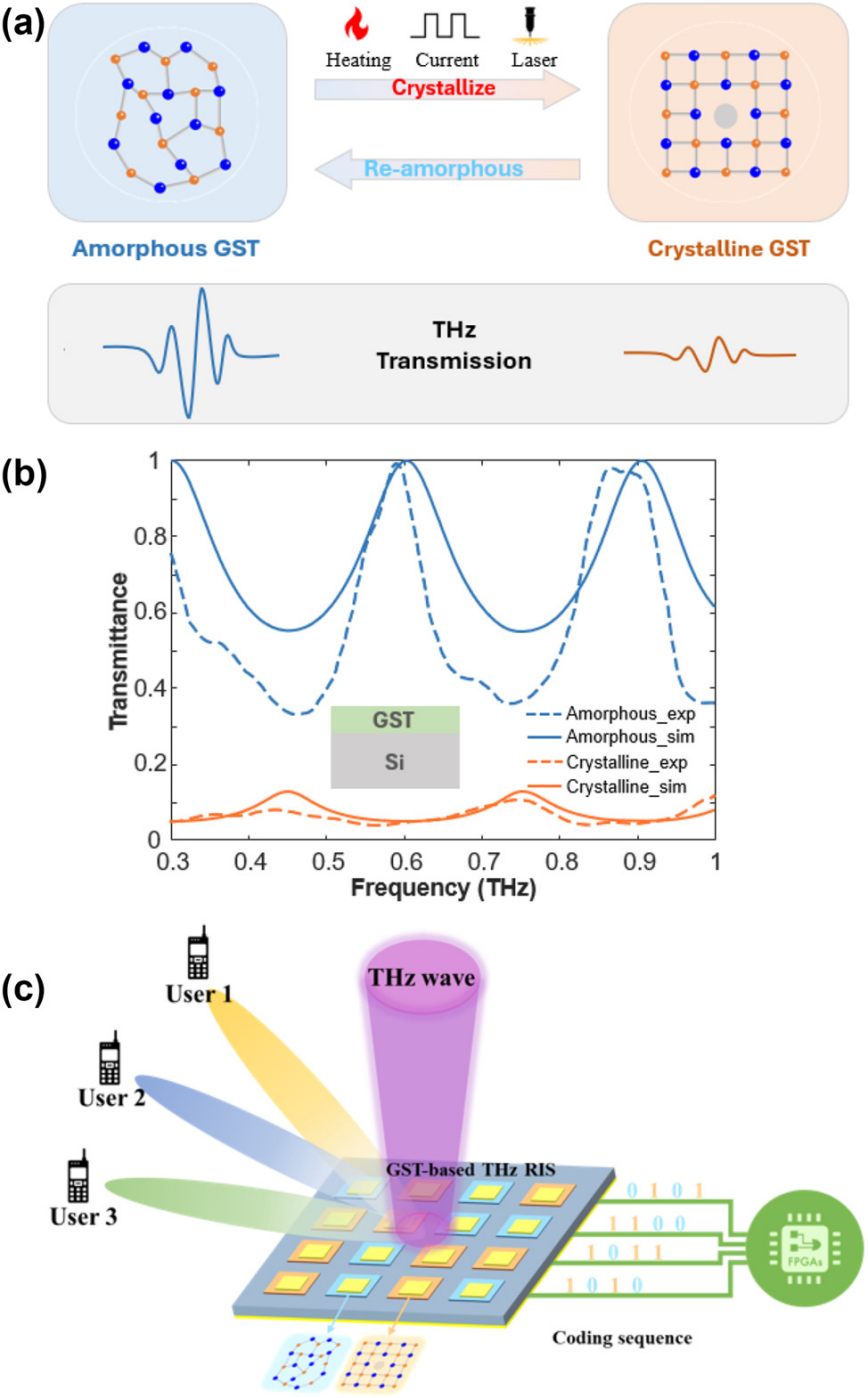
Active modulation of THz wave with phase-change GST films. (a) Phase states change with thermal, electrical, and optical excitations and the corresponding THz responses. (b) Measured and simulated transmission of a GST film on a p-type silicon substrate at amorphous and crystalline states. The film thickness is 300 nm. (c) Schematic diagram of GST-based THz RIS.

To enable THz RIS, several active control materials have been investigated, including graphene, liquid crystals, and phase-change materials [10]. Ge_2_Sb_2_Te_5_ (GST), a chalcogenide phase-change material, is notable for its vast conductivity and permittivity variation and picosecond switching speed between amorphous and crystalline states [11]–[13], as shown in Figure 1(a) and (b). A comparison of the modulation performances for THz MTMs with graphene, and vanadium dioxide (VO_2_), is methodically expounded in Table. 1 [14]–[16], which highlights the advancement of GST in terms of modulation deep and speed. Furthermore, its non-volatile characteristics show significant potential for developing advanced THz RIS systems with integrated memory functions and low power consumption.

**Table 1:** The comparison of modulation performances with graphene, VO_2_, and GST for THz MTMs.

Tuning element	Graphene	VO_2_	GST
Tuning mechanism	Fermi level	Photothermal	Photothermal
Control method	Bias voltage	Laser pluses	Laser pluses
Frequence (THz)	0.8	0.864	0.5–0.8
Modulation depth	80 %	54 %	100 %
Modulation speed	3000 ps	30 ps	19 ps
Material property	Volatile	Volatile	Non-volatile
Ref.	[14]	[15]	[16]

This work briefly scrutinizes the recent advancements of active THz MTMs with GST in the last three years, spotlights the external stimuli facilitating GST phase transitions, discusses their constraints and unsolved problems, and forecasts a progressive trajectory toward the non-volatile, multifunctional GST-based THz RIS in the rising 6G communication systems.

## 2 Active THz MTMs with GST

**Figure 2:**
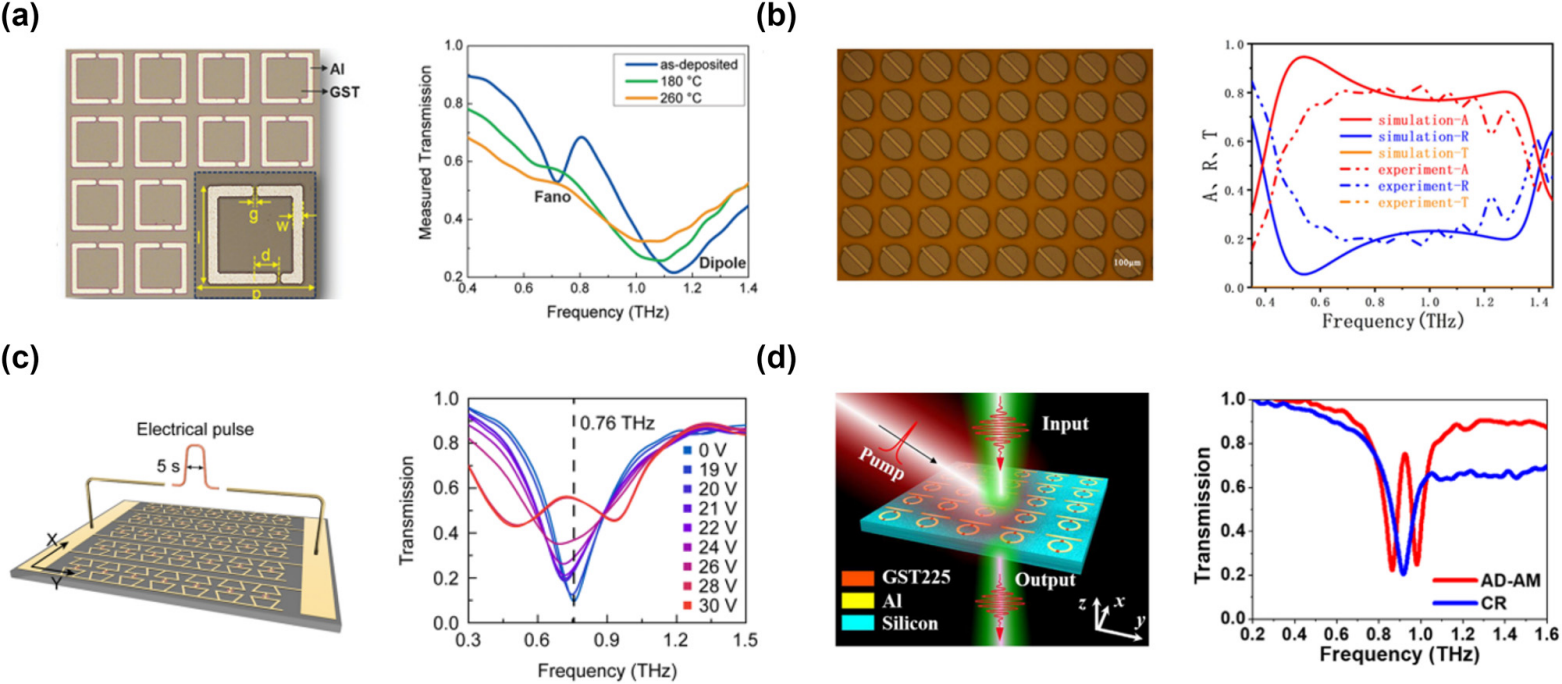
Active THz MTMs with GST. (a) Active modulation of THz SRRs with GST film [16]. (b) Thermally switchable bi-functional metasurface [17]. (c) Electrically controlled multilevel modulation [18]. (d) EIT metasurface [19]. (a) Reproduced with permission [16]. Copyright 2019, Wiley-VCH. (b) Reproduced with permission [17]. Copyright 2022, Wiley-VCH. (c) Reproduced with permission [18]. Copyright 2022, American Chemical Society. (d) Reproduced with permission [19]. Copyright 2022, Wiley-VCH.

GST was initially proposed for actively modulating THz MTMs in 2019 [16], as illustrated in Figure 2(a). In this configuration, GST films, sputter-deposited onto quartz substrates, enable non-volatile control of Fano and dipole resonant modes in split-ring resonators (SRRs), achieving spatial and temporal selectivity. Various phase-change approaches have been investigated in this work, including thermal annealing, current pulse, and optical pumping. Since then, GST has attracted considerable interest in the active MTMs at THz frequencies.

### 2.1 Thermal control

Thermal control with hot plate annealing and resistive micro-heater is typically considered as the primary method for the phase transition in GST films. J. Chen et al. introduced a thermally switchable bi-functional metasurface integrated with GST in 2022 [17], as depicted in Figure 2(b), enabling a polarization conversion ratio (PCR) exceeding 90 % from 0.6 to 1.15 THz. Once the GST conductivity reaches 1 × 10^5^ S/m, the metasurface can function as a broadband absorber from 0.44 to 1.34 THz. In 2023, M. Lian et al. reported a flexible bilayer metamaterial consisting of a square disk and outer double SRRs, achieving an impressive PCR of over 0.7 from 0.53 to 1.25 THz [20]. The general challenge of thermal treatments is the non-uniform intermediate states with varying proportions of amorphous and crystalline phases induced by temperature fluctuations [21]. Non-uniformity may defect the robustness and repeatability of the MTM devices, particularly for the THz MTMs with relatively large scales. In this regard, microheaters should be carefully designed with uniform annealing. Furthermore, the transition speed of thermal methods has yet to be improved to meet the demands of high-speed applications.

### 2.2 Current modulation

The urgent demands for high integration level and high phase-change speed inspire growing interest in exploring electrical control methods of GST films. In 2022, X. Chen et al. investigated a hybrid plasmonic dimer with GST electrical switching, which manipulates both the resonant amplitude and frequency of THz waves by applying multilevel current pulses [18], as shown in Figure 2(c). In addition to the THz devices, visible and infrared devices with electrical GST modulation have been investigated more frequently [8], [22]. However, the same challenge, i.e., filamentation, remains to be solved in both optical and THz realms. It can interfere with uniform crystallization under current pulses and, thus, limits film uniformity and large-area applications.

### 2.3 Optical excitation

As a wireless approach, optical pumping is an alternative avenue for ultrafast phase transition in GST, the corresponding response time could be down to the order of picoseconds. In 2021, a GST-integrated metasurface on a polyimide substrate was studied, enabling Fano resonance control through optical stimulus [23]. In 2022, a reconfigurable electromagnetically induced transparency (EIT) metasurface at 0.92 THz was reported, consisting of a functional resonator integrated with GST film [19], which achieves a reversible switching under nanosecond laser pulse excitation, as shown in Figure 2(d). Additionally, H. Zhu et al. systematically investigated the thermal equilibrium process and ultrafast dynamics of GST with femtosecond laser pulses in 2022 [24]. Although optical pumping with femtosecond lasers provides high phase-change speed, the auxiliary and bulky laser sources are not friendly for the array applications. Another limitation is that optical excitation is struggling to individually modulate each pixel of a large MTM array with a single illumination.

## 3 Programmable THz MTMs with GST for RIS

In 2022, programmable THz metasurfaces with GeTe films were reported [25], which highlight the versatility and potential of chalcogenide phase change materials in digital THz wave manipulation, i.e., a key capability of RIS. In 2023, theoretical simulations of GST-based digital surfaces were reported, demonstrating the potential of GST for precisely controlling beam direction and modulation amplitude [26]. The proposed models and programmable concepts contribute to advance the GST-based RIS implementation in the future 6G communications.

## 4 Conclusion and prospective

The progression towards 6G MIMO communication heralds a significant shift in wireless network capabilities, emphasizing ultra-fast, reliable, and energy-efficient operations. RIS utilizes digitally controlled MTMs to dynamically manipulate electromagnetic waves, achieving a more flexible and controllable wireless transmission environment. However, current RIS systems are typically designed with PIN and varactor diodes with commercial packaging, which cannot directly mitigate to THz frequencies due to their package size and cut-off frequencies. Their beam switching time is expected to be below 500 ns for most application scenarios. With the benefit of non-volatile phase transition properties, rapid state-switching capabilities, and multi-level modulation, GST shows significant advantages over the other materials in the THz spectrum. The incorporation of GST into RIS is instrumental in overcoming the cost and energy problems in the advanced THz communication systems. However, several challenges warrant careful scrutiny in the thermal, electrical, and optical approaches. Thermal annealing exhibits intrinsic limits in phase transition rate and modulation speed. Electrical methods are pivotal for compact circuit integration and multilevel control, however, which may suffer from filamentation and heating uniformity. Optical pumping offers rapid phase transition in the focal spot. However, the laser sources are bulky, complex, and expensive for practical applications.

Despite the aforementioned challenges, GST-tuned THz MTMs show great promise in the MIMO systems of the 6G communication due to their large modulation range, high switching speed, and non-volatile and continuous modulation. Compared to the other active stimuli at THz frequencies, such as VO_2_ (it requires 1.125 W of sustained electrical power or a switching optical power of 5 mJ/cm^2^ for ultrafast phase changes within 30 ps [15]), GST stands out with its much lower switching power, i.e., 0.6366 mJ/cm^2^ for effective non-volatile phase changes within 19 ps [16]. Micro-heater and current pulses could be directly integrated with field programmable gate arrays (FPGAs), which could be functional digitally and programmably, as shown in Figure 1(c). In this case, GST may enable THz RIS as the substitute for the classic PIN diodes at microwave frequency. Furthermore, optically addressed GST RIS can be manipulated with laser beams or fiber arrays instead of complicated multilayer bias networks. Most notably, the non-volatile GST approach addresses low power consumption, which is one of the key constraints for 6G commercialization. Further research directions may include hybrid modulation strategies with thermal, electrical, and optical methods, dynamic beamforming with memories, and ultra-high-speed THz communication with GST RIS.
